# Does Metoclopramide Reduce Variceal Pressure?

**DOI:** 10.1155/1992/60853

**Published:** 1992

**Authors:** R. A. J. Spence

**Affiliations:** Belfast City Hospital Lisburn Road Belfast BT9 7AB Ireland


out, should give a better evaluation of its diagnostic and therapeutic value in patients who are to undergo biliary surgery.

Professor P.C. Bornman
Department of Surgery
University of Cape Town Medical School
Observatory 7925
Cape Town
South Africa

### REFERENCES

1. Neoptolemos, J.P., Carr-Locke, D.L. and Fossard, D.P (1987) Prospective randomised study of pre-operastive endoscopic sphincterotomy versus surgery alone for common bile duct stones. *British Medical Journal*, **294**, 470–474
2. Neoptolemos, J.P., Davidson, B.R., Shaw, D.E. *et al.*, (1987) Study of common bile duct exploration and endoscopic sphincterotomy in a consecutive series of 438 patients. *British Journal of Surgery*, **74**, 916–921
3. Muller, B.M., Kozarek, C.A., Ryan, J.A. Jr. *et al.*, (1988) Surgical versus endoscopic management of common bile duct stones. *Annals of Surgery*, **207**, 135–141
4. Pappas, T.N., Slimane, T.B. and Brookes, D.C. (1990) 100 Consecutive common duct explorations without mortality. *Annals of Surgery*, **211**, 260–262
5. Van Stiegmann, G., Pearlman, N.W., Goff, J.S., Sun, J.H. and Nortone, C.W. (1989) Endoscopic cholangiography and stone removal prior to cholecystectomy. *Archives of Surgery*, **124**, 787–790

### DOES METOCLOPRAMIDE REDUCE VARICEAL PRESSURE?

### ABSTRACT

*Kleber, G., Sauerbruch, T., Fischer, G., Geigenberger, G., Paumgartner, G. (1991) Reduction of transmural oesophageal variceal pressure by metoclopramide. Journal of Hepatology;* **12**: 362–366.

**In nineteen patients with portal hypertension and oesophageal varices, transmural variceal blood pressure was determined endoscopically by direct puncture of the varices before and after intravenous administration of 20 mg metoclopramide or placebo. No change in pressure was observed after placebo (mean difference –1.3 ± 24.5%, N.S.), however, metoclopramide reduced the pressure by 17.6 ± 18.6% (*p* = 0.02). Our results suggest that metoclopramide may be beneficial for the prevention of treatment of variceal haemorrhage.**

### PAPER DISCUSSION

KEY WORDS: Portal hypertension, metoclopramide, portal pressure, oesophageal

This paper reports a study of 19 patients who had portal hypertension with oesophageal varices and in whom transmural variceal pressure was assessed. This was measured before and after intravenous administration of Metoclopramide or placebo.

The idea of pharmacological manipulation of the lower oesophageal sphincter in patients with portal hypertension is not new. In 1978, Miskowik suggested that the lower oesophageal sphincter affected blood flow in the submucosal oesophageal varices[1].

It seems reasonable from anatomical studies that manipulation of the lower oesophageal sphincter could affect the blood flow in distal oesophageal varices. The detailed anatomy of the lower oesophageal veins was first described by Butler in 1951[2]. However, one of the most important pieces of anatomical work came from Brazil with the study of de Carvalho[3]. He first drew attention to the fact that the veins in the distal part of the oesophagus, namely those veins lying near the oesophago-gastric junction and therefore near the oesophageal sphincter, seem to lie superficially, whereas the veins in the stomach and the veins in the more proximal oesophagus tended to lie more deeply in the submucosa. During the past decade several complementary anatomical investigations using modern techniques have been published. A computer assisted image analysis study showed that the veins in the distal oesophagus were lying superficially in the lamina propria, whereas in the stomach and in the proximal oesophagus the veins lay chiefly in the submucosa[4].

This anatomical work proposed that the distal oesophagus (2cm to 5cm) is the vulnerable area where varices are more prone to rupture[5]. Subsequent complementary studies have been published demonstrating a constant perforating vein at 36cm using Doppler ultrasound. This further indicated that the lower oesophageal sphincter in association with the local venous anatomy may have a role to play in the pathogenesis of the site of variceal rupture[6].

A study in Japan[7] has confirmed the superficial nature of the veins in the distal oesophagus. Further anatomical studies from Capetown described four layers of veins in the oesophagus (1) intraepithelial vascular channels; (2) superficial venous plexus; (3) deep venous plexus and (4) extrinsic veins which communicated with the stomach veins[8].

Studies from King's College Hospital have also looked at the venous anatomy in the lower oesophagus, using resin casting, and the authors have described four zones: (a) gastric zone with veins in a longitudinal venous distribution; (b) palisade zone with parallel vessels mainly in the lamina propria; (c) perforating zone with "treble clef" veins and finally (d) a truncal zone with deep veins[9]. Further work from Japan[10] has described findings similar to those of the King's College Group.

It therefore seems clear that the critical area of rupture of varices appears to be the lower 2cm to 5cm of the oesophagus and this may be related to the now well described anatomical features of the venous drainage of this area which teleologically may be related to the presence of the lower oesophageal sphincter mechanism[11].

Fifteen years ago Cohen and his colleagues showed that metoclopramide gave a dose-related increase in the lower oesophageal pressure both in response to oral and intravenous administration of the drug[12]. However, it should be noted that the accepted oral dose at that time was 10mg and this gave only a slight increase in the lower oesophageal pressure which was not consistent in all of their patients. Subsequently the Danish group in a very small series of patients (6) and using a rather crude technique of measuring blood flow showed that metoclopramide decreased the flow to the varices in five patients, and they related this to the increase in the lower oesophageal sphincter pressure produced by the drug[13].

Subsequently Lunderquist, using percutaneous transhepatic portography in patients with portal hypertension and oesophageal varices showed that a number of drugs could decrease variceal blood flow through the lower oesophagus. Although the number of patients was very small the group showed that vasopressin did not change the variceal blood flow. However, pentagastrin, presumably with its effect of increasing the lower oesophageal sphincter pressure, interrupted the blood flow, through the varices, in four of eight patients[14].

In studies using azygos blood flow measurements in a small group of six patients from Lebrec's group, while an increase in lower oesophageal sphincter pressure induced by domperidone was found, azygos blood was unaltered[15]. However, in a larger series of patients contrary data from the Barcelona group has been published. In 33 patients with azygos venous blood flow measurements the administration of both metoclopramide and domperidone caused a significant reduction in azygos blood flow, compared to a placebo group. The authors concluded that this was a local effect of the drugs in increasing the lower oesophageal sphincter pressure as all the other parameters of portal pressure, hepatic blood flow and cardiac output remained unaltered[16].

Transmural oesophageal variceal pressure has been shown to be easily measured by direct puncture of oesophageal varices[17]. Subsequently, the Sheffield group showed that pharmacological constriction of the lower oesophageal sphincter did reduce intravariceal pressure from a mean of 23mm Hg to 4mm Hg and these data were significant. In a subsequent small trial in Egyptian patients who were actively bleeding from varices in the lower 2cm of the oesophagus there appeared to be benefit in giving metoclopramide[18].

The current paper from Munich shows that 20mg of intravenous metoclopramide significantly reduced intravariceal pressure, compared to a placebo. The two groups in the study were comparable but nonetheless of a total of 50 patients being entered into the study, 31 of these had to be excluded, mainly because of unsatisfactory pressure recordings. Some of these unsatisfactory recordings were due to the varices being small and therefore difficulty may have occurred in inserting the needle into the varix. This could alter the results as it has been well shown that the larger varices tend to bleed more than smaller ones[19]. Furthermore, the numbers in the study were relatively small with ten and nine in each group. In addition, there were two patients, one in each group, who appeared to sustain a remarkably large drop in pressure and again this could alter the results.

Nonetheless, the data from the paper under review and that of other data in the literature indicate that overall there is probably a reduction in intravariceal pressure by metoclopramide. However, although the data show a reduction in intravariceal pressure with metoclopramide whether or not this can be translated into clinical benefit is currently unclear.

A further study from the Barcelona group has shown no benefit with either domperidone or metoclopramide given intravenously in patients who have had bleeding from oesophageal varices. In a controlled study there is no difference in re-bleeding rate compared to a placebo group, although the criteria for diagnosing re-bleeding from the varices in this study could be criticised[20].

In summary it seems likely that increasing the lower oesophageal sphincter pressure by drugs such as metoclopramide increases the in-flow resistance into the varices and may shift the collateral blood flow into the peri-oesophageal veins[21].

There is therefore a sound anatomical and physiological basis for this hypothesis but further clinical controlled trials are required.

### REFERENCES

1. Miskowik, J. (1978) How the lower oesophageal sphincter affects submucosal oesophageal varices. *Lancet*, **2**, 1284–1285
2. Butler, H. (1951) The veins of the oesophagus. *Thorax*, **6**, 276–296
3. de Carvalho, C.A.F. (1966) Sur L'angio architecture veineuse de la zone de transition oesophago-gastrique et son interpretation fonctionell. *Acta Anat*, **64**, 125–161
4. Spence, R.A.J. (1984) The venous anatomy of the lower oesophagus in normal subjects and in patients with varices — an image analysis study. *Brit. J. Surg.*, **71**, 739–744
5. Spence, R.A.J. (1991) Venous drainage of the oesophagus. *Gullet*, **1**, 163–165
6. McCormack, T.T., Rose, J.D., Smith, P.M. and Johnson, A.G. (1983) Perforating veins and blood flow in oesophageal varices. *Lancet*, **2**, 1442–1444
7. Noda, T. (1984) Angioarchitectural study of esophageal varices. *Virch. Arch.*, **404**, 381–392
8. Kitano, S., Terblanche, J., Kahn, D. and Bornman, P.C. (1986) Venous anatomy of the lower oesophagus in portal hypertension — practical implications. *Brit. J. Surg.*, **73**, 525–531
9. Vianna, A., Hayes, P.C., Moscoso, G., Driver, M., Portmann, B., Westaby, D. and Williams, R. (1987) Normal venous circulation of the gastro-oesophageal junction. *Gastroenterology*, **93**, 876–889
10. Hashizume, M., Kitano, S., Sugimachi, K. and Sueishi, K. (1988) Three dimensional view of the vascular structure of the lower oesophagus in clinical portal hypertension. *Hepatology*, **8**, 1482–1487
11. Arakawa, M. and Kage, M. (1991) The anatomy and pathomorphology of oesophageal varices in portal hypertension. Clinical and physiological aspects. Eds. Okuda, K. and Benhamou, J-P. Springer-Verlag; 415–428
12. Cohen, S., Morris, D.W., Schoen, H.J. and Dimarino, A.J. (1976) The effect of oral and intravenous metoclopramide on human lower esophageal sphincter pressure. *Gastroenterology*, **70**, 484–487
13. Miskowik, J., Burcarth, F. and Jensen, L.I. (1981) Effect of a lower oesophageal sphincter on oesophageal varices. A portographic study. *Scand. J. Gastro.*, **16**, 957–960
14. Lunderquist, A., Alwmark, A. and Gullstrand, P. *et al.* (1983) Pharmacologic influence on esophageal varices — a preliminary report. *Cardiovasc. Intervent. Radiol.*, **6**, 65–71
15. Braillon, A., Capron-Chivrac, D. and Valla, D. *et al.* (1986) Domperidone — induced increase in lower oesophageal sphincter pressure does not affect azygos blood flow in patients with cirrhosis. *Scand. J. Gastro.*, **21**, 1080–1082
16. Mastai, R., Grande, L. and Bosch, J. *et al.* (1986) The effects of metoclopramide and domperidone on azygos venous blood flow in patients with cirrhosis and portal hypertension. *Hepatology*, **6**, 1244–1247
17. Kleber, G., Sauerbruch, T., Fischer, G. and Paumgartner, G. (1988) Somatostatin does not reduce oesophageal variceal pressure in liver cirrhotics. *Gut*, **29**, 153–156
18. Hosking, S.W., Doss, W., El-Zeiny, H. *et al.* (1988) Pharmacological constriction of the lower oesophageal sphincter — a simple method of arresting variceal haemorrhage. *Gut*, **29**, 1098–1102
19. The North Italian Endoscopic Club. (1988) Prediction of the first variceal haemorrhage in patients with cirrhosis of the liver and oesophageal varices. *New Eng. J. Med.*, **319**, 983–989

20. Feu, F., Mas, A., Bosch, J. *et al.* (1988) Domperidone or metoclopramide versus placebo in the prevention of early variceal re-bleeding in cirrhosis. A prospective randomised trial. *J. Hepatology*, **7**, 531
21. Navasa, M., Bosch, J., Rodes, J. (1991) Pharmacological agents and portal hypertension. *In Portal Hypertension*, Ed. Okuda, K., Benhamou, J-P, Springer-Verlag, 35–49

R.A.J. Spence
Consultant Surgeon
Belfast City Hospital
Lisburn Road
Belfast BT9 7AB
Northern Ireland

### SOMATOSTATIN FOR BLEEDING OESOPHAGITIS OR ULCERATION AFTER SCLEROTHERAPY FOR OESOPHAGEAL VARICES

### ABSTRACT

*Jenkins, S.A., Shields, R., Jaser, N., Ellenbogen, S., Makin, C., Naylor, E., Newstead, M., Baxter, J.N. (1991) The Management of gastrointestinal haemorrhage by somatostatin after apparently successful endoscopic injection sclerotherapy for bleeding oesophageal varices. Journal of Hepatology;* **12**: 296–301.

**Twenty-two patients who experienced a severe haemorrhage from either oesophagitis ($n = 8$) or ulcers ($n = 14$) following injection sclerotherapy of their oesophageal varices were treated with intravenous administration of somatostatin (250 μg/h). Somatostatin was effective in controlling haemorrhage and preventing rebleeding in all eight patients bleeding from oesophagitis and in 12 of the 14 patients bleeding from oesophageal ulcers. In two patients with ulcers, haemorrhage persisted despite two periods of concominant balloon tamponade and somatostatin infusion and bleeding was eventually controlled by repeated hourly bolus injections of the hormone for 24 h superimposed on the continuous infusion. The results of this study suggest that somatostatin is an effective and safe treatment for the control of bleeding from either oesophagitis or ulcers following injection sclerotherapy of oesophageal varices.**

